# The Need for More Learning Opportunities in Real-Time Ultrasonography for Undergraduate Medical Students: A Call for Pre-clerkship Electives

**DOI:** 10.1007/s40670-024-02069-x

**Published:** 2024-05-28

**Authors:** Genesys Santana, Sergio Bustamante, Erik Kraenzler

**Affiliations:** 1 School of Medicine, Case Western Reserve University, Cleveland, OH USA; 2 Department of Cardiothoracic Anesthesiology, Cleveland Clinic Foundation, Cleveland, OH USA

## Abstract

Medical education is at a point of transition in which it must reform to meet the demand of healthcare providers competent in ultrasonography. This article recommends the pre-clerkship elective infrastructure as a method for medical schools to begin prototyping content and modes of delivering ultrasound education. Doing so will prepare medical students earlier in their careers to use and build upon their knowledge of ultrasound during clerkships and as residents.

In an age in which medical knowledge doubles every few months with technology advancing fast, healthcare practitioners must constantly update their skillsets. As a result, curricula in undergraduate and graduate medical education must adapt to meet the demand, or risk becoming irrelevant. This is exemplified by the increasing importance of the use of ultrasound technologies as procedural and diagnostic tools in the clinical arena creating the need to increase ultrasound education [1–3].

The medical education community has responded in various ways, with some medical schools now integrating ultrasound education in their core curriculum [4, 5]. A study conducted in 2021 found that 168 medical schools had ultrasound curriculum [6]. At Case Western Reserve University School of Medicine, students receive ultrasound education on specific anatomical topics during their pre-clerkship, as part of a tripartite curriculum known as Gross Anatomy, Radiology and Living Anatomy (GARLA) [7].

Courses like GARLA symbolize progress, but they leave wanting more, with participating students still having scant exposure to ultrasound. Additionally, they emphasize point-of-care ultrasound (POCUS) diagnostics, while negating to include the procedural applications of ultrasound, like real-time ultrasonography for guided vascular procedures, routinely performed in multiple fields, including anesthesiology, vascular surgery, and interventional radiology.

We approach this topic from either side of the gap—as an undergraduate medical student and attendings who train graduate medical students. After reflecting on our personal experiences and reviewing peer-reviewed studies with similar concerns, we firmly believe ultrasound education needs to evolve to be more ubiquitous—included in the curricula of all medical schools—and more encompassing—teaching both procedural and diagnostic applications of ultrasonography.

Without changes, medical students will continue to be unprepared to fulfill their future roles as physicians who can achieve patient safety through ultrasound-guided procedures. Here, we invite the medical education community to ask the following: (1) Why is there a discrepancy between the demand for clinicians who know how to use ultrasound for procedures and not just diagnostics, and what undergraduate medical education supplies? and most importantly, (2) what are some short- and long-term solutions that the medical education community can implement to bridge this gap?

The deficiency in ultrasound education is attributable to multiple factors that plague undergraduate medical education in general. Like other forms of higher education, undergraduate medical education is at a crossroads. Whether because of conservatism, risk aversion, lack of funding, or time constraints, institutions of medical education often take too long to adapt technological and methodological innovations in their curricular content [8]. Students are questioning whether existing curricular paradigms really set them up for success, especially during the pre-clerkship years [9, 10].

This issue is compounded by the general dearth of clinical skills training in undergraduate medical education, during both pre-clerkship and clerkship years. The impending integration of artificial intelligence in medical practice makes it more important than ever for medical practitioners to acquire fine motor and procedural skills to be marketable [11]. Yet, medical students have low self-reported competence in many procedural skills, leaving them feeling unprepared for clerkships [12, 13]. Unfortunately, existing literature suggests this is not addressed during the clerkship years. There is a marked prevalence of residency program directors whose expectations of how many and what types of procedural skills medical school graduates can perform independently exceed what they can deliver at residency onset. This mismatch of expectations and experience has important implications for patient safety [14].

Integrating diagnostic and procedural applications of ultrasound in pre-clerkship curriculum provides a two-fold benefit. It is an opportunity for undergraduate medical education to make itself more relevant and valuable to students by providing a service (equipment and expert guidance) that outside sources cannot equate. Also, it will equip students with increasing confidence and a strong foundation in procedural skills they can build on during their clerkship years. By the time they reach residency, they will be prepared to provide high-quality care and safety.

The long-term solution is for all medical schools to integrate ultrasound education in their core curriculum and for existing programs to expand their coverage of ultrasound topics. Although this will take time, we propose that institutions provide students with simulation courses on real-time ultrasonography procedural techniques organized using a pre-clerkship elective infrastructure. We see pre-clerkship electives as alternative short-term solutions that offer students with valuable learning opportunities to supplement the existing core curriculum. A previous study demonstrated that students who complete electives in procedural skills report being more comfortable with and demonstrate improved skills in performing procedures by the end of the course [12]. It also provides institutions with an incubator in which they can create, test, and optimize different content and modalities of delivery—before offering it to the larger population of medical students through the core curriculum.

In many ways, it is part of a larger legacy in the history of ultrasound in undergraduate medical education, in line with the ultrasound interest groups that are predecessors of many of today’s formal ultrasound curriculum in medical schools [15]. Through them, students and faculty make clear there is a need and demand for ultrasound education. Most importantly, students take ownership of their education, and in partnership with their faculty mentors, demonstrate to the administration how it can be done.

In this spirit, together we established a pre-clerkship elective at Case Western Reserve University School of Medicine. It inaugurated in the 2022–2023 academic year and is titled ACCM 9000C Real-Time Ultrasonography Techniques: A Simulation Based Training. Enrollment is voluntary and open to all first- and second-year medical students. It serves to supplement the curriculum of Gross Anatomy, Radiology and Living Anatomy (GARLA) [7]. To date, 13 students completed the course and there are 15 in the current 2023–2024 cohort. We hope to optimize this model that schools can later adopt.

Medical education must adapt to changes in clinical practice so that students are prepared for their future roles as physicians. Currently, this means that ultrasound education must be integrated into the existing curriculum. We hope the infrastructure of a pre-clerkship elective presents an opportunity for medical schools to begin doing so.
